# First report of *Anaplasma ovis* in pupal and adult *Melophagus ovinus* (sheep ked) collected in South Xinjiang, China

**DOI:** 10.1186/s13071-018-2788-6

**Published:** 2018-04-19

**Authors:** Li Zhao, Bo He, Kai-Rui Li, Fei Li, Lu-Yao Zhang, Xian-Qiang Li, Yong-Hong Liu

**Affiliations:** 1 College of Animal Science, Tarim University, 705 Hongqiao South Road, Aral, 843300 People’s Republic of China; 2 Key Laboratory of Tarim Animanl Husbandry Science and Technology of Xinjiang Production & Construction Corps, 705 Hongqiao South Road, Aral, 843300 People’s Republic of China; 3 Animal Loimia Controlling and Diagnostic Center of Aksu Region, Friendship Road, Aksu, 843000 People’s Republic of China

**Keywords:** *Melophagus ovinus*, *Anaplasma ovis*, China

## Abstract

**Background:**

*Melophagus ovinus* (sheep ked) is a blood-feeding ectoparasite that belongs to the family Hippoboscidae (Diptera: Hippoboscoidea) and mainly parasitizes sheep. The life-cycle of *M. ovinus* consists of three stages: larva, pupa and adult. It has a worldwide distribution and has been found in four provinces of China, especially South Xinjiang. In addition to causing direct damage to animal hosts, *M. ovinus* serves as a vector for disease transmission. In this study, our aim was to investigate the presence of *Anaplasma* spp. in pupal and adult *M. ovinus*.

**Methods:**

A total of 93 specimens (including eight pupal specimens) of *M. ovinus* collected in South Xinjiang were selected for isolation of genomic DNA, followed by PCR amplification and sequencing of the *msp4* gene of *Anaplasma* spp. The sequences were analyzed in MEGA 7.0 software and *via* online BLAST.

**Results:**

PCR and sequencing results showed that all the specimens collected in 2013 were free of *Anaplasma* spp., whereas three and 25 specimens (including five pupal specimens) collected in 2016 and 2017, respectively, tested positive for *Anaplasma* spp. The analysis of 24 *msp4* gene sequences (from four pupal specimens) confirmed the presence of *A. ovis* in *M. ovinus* specimens collected in South Xinjiang, China. The detected *A. ovis* isolates belong to Genotypes II and III.

**Conclusions:**

To the best of our knowledge, this is the first report of the detection of *A. ovis* DNA in pupal *M. ovinus*, confirming the vertical transmission of *A. ovis* in *M. ovinus* and the potential of *M. ovinus* to serve as a vector for *A. ovis*.

## Background

**Fig. 1:**
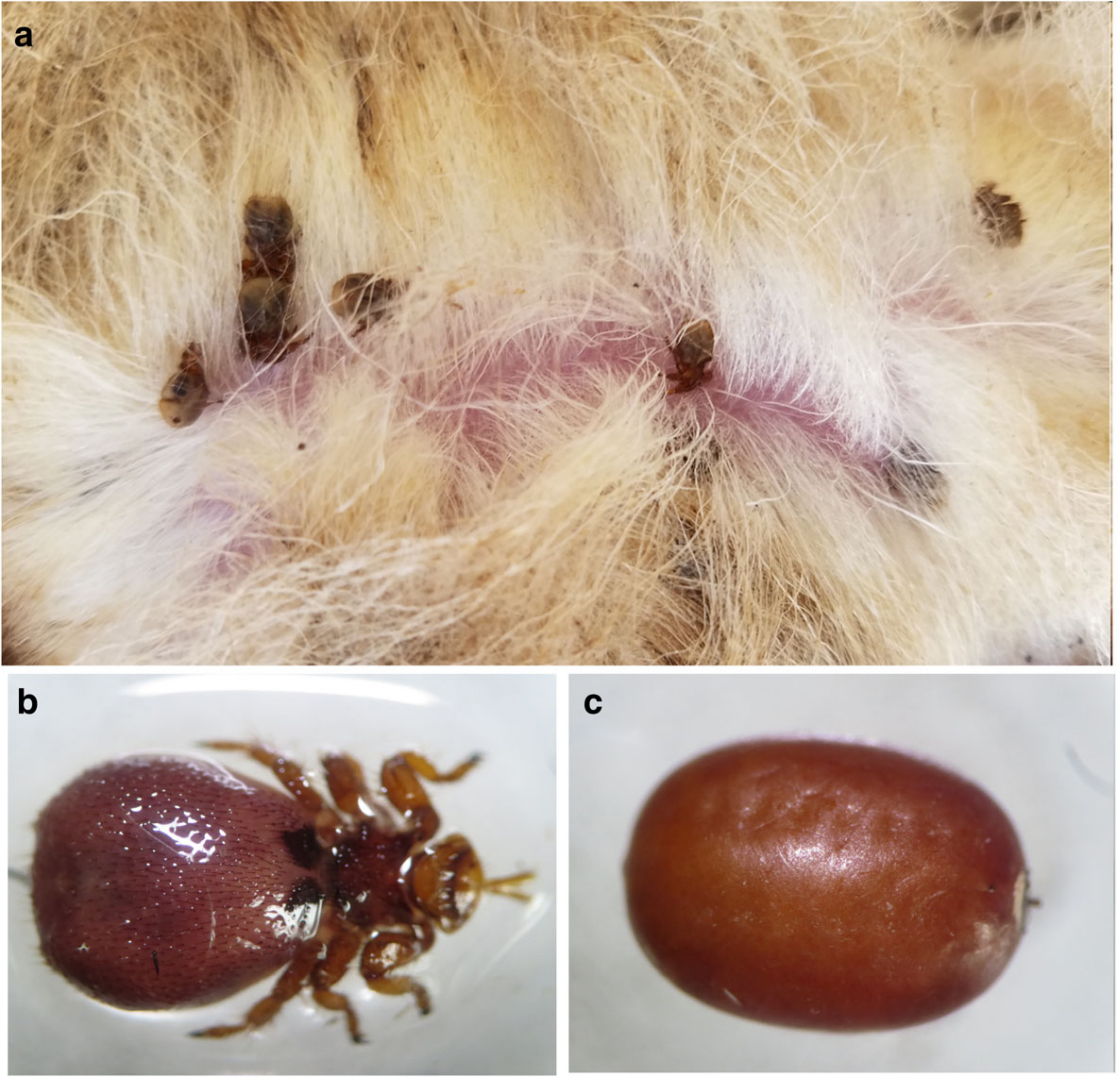
Sheep from Yahazhen of Kuqa in Xinjiang in June 2017. **a.** *M. ovinus* parasitizes sheep in fur. *M. ovinus* could be found in the fur-covered area all over the body, including ears (and behind the ears), neck, chest, abdomen, back, breech, legs, and tail. **b.** Adult *M. ovinus*. **c.** Pupal *M. ovinus*

*Melophagus ovinus* (sheep ked), is a blood-feeding ectoparasite that belongs to the family Hippoboscidae (Diptera: Hippoboscoidea) and has significant economic effects [1, 2]. *Melophagus ovinus* (Fig. 1a, b) is an approximately 4–6 mm long wingless fly with a small head, strong and sharp mouthparts, an oval or round abdomen, dense bristles on the body surface, and three pairs of legs tipped with claws [2, 3].

The life-cycle of *M. ovinus* consists of three stages: larva, pupa (Fig. 1c) and adult [1, 4]. Six to eight days after mating, the female fly produces larvae that adhere to the body surface of hosts and are ready to pupate into brown pupae within 6–12 hours. After 19–30 days, the pupae develop into adults, which parasitize the body surface of sheep [1].

*Melophagus ovinus* is widely distributed and has been found in many European, African, Asian, Oceanian, and North American countries [2]. Until now, *M. ovinus* has been reported to parasitize only sheep and Tibetan antelopes in Xinjiang [2, 5], Qinghai [2, 6] and Gansu [3, 7] in China. Additionally, adult or pupal *M. ovinus* specimens have been detected on imported sheep and sheep skin and wool during port-quarantine in certain areas of China [8, 9].

*Melophagus ovinus* mainly parasitizes sheep but has also been found to have an expanded host range, which includes goats [10], rabbits (*Oryctolagus cuniculus*) [1], dogs [11], wild animals [Tibetan antelope [6], European bison (*Bison bonasus*) [12], and red foxes (*Vulpes vulpes*) [13]] and humans [11]. It is mainly directly transmitted among sheep during transportation, mixed grazing, sheep crowding, and direct contact between ewes and lambs [14] as well as indirectly transmitted through bedding and tools [1, 7].

Upon infection, *M. ovinus* bites and feeds on the blood of sheep, leading to irritation, inflammation, anemia, and subsequently loss of wool, as well as skin damage due to biting, kicking, and rubbing of invaded sites. These actions in turn cause secondary microbial infections or myiasis. Additionally, *M. ovinus* infestation leads to weight gain and attenuates wool production: effects that compromise the quality and yield of wool as well as the value of sheep skin [1–4]. Moreover, *M. ovinus* serves as an insect vector (or potential vector) for pathogens and has been reported to be responsible for the transmission of e.g. *Trypanosoma melophagium* [14], *Anaplasma ovis* [15], blue-tongue virus [16], *Bartonella schoenbuchensis*, *Bartonella chomelii* [17], *Bartonella melophagi* [4, 18] and other *Bartonella* spp. [19] worldwide. In China, *Bartonella garinii*, a *B. valaisiana*-like group [5], *Rickettsia raoultii*, *R. slovaca* [2], *Bartonella* spp., *Arsenophonus*, *Wolbachia* [3, 7], *Enterobacter*, *Acinetobacter*, *Halomonas*, *Shewanella*, *Bacillus* and *Staphylococcus* [3] have also been detected in *M. ovinus*. In summary, *M. ovinus* causes huge economic losses either directly or indirectly.

*Anaplasma ovis* is an obligate intraerythrocytic pathogen infecting sheep, goats, and some wild ruminants [20–25]. It belongs to the genus *Anaplasma* (Rickettsiales: Anaplasmataceae), which has been recently confirmed to include other species responsible for anaplasmosis, such as *A. marginale*, *A. phagocytophilum*, *A. centrale*, *A. bovis*, *A. platys* and *A. capra*. Anaplasmosis is an important veterinary and public health issue globally that leads to serious economic losses [25, 26].

The major surface protein 4 (*msp4*) gene of *Anaplasma* spp. is highly conserved among many strains [20, 27]. It has been demonstrated that PCR amplification of the *msp4* gene has a high diagnostic value for the differential detection of *A. ovis* [20, 22, 28]. The *msp4* gene has also been applied to genetic characterization and phylogenetic studies of *Anaplasma* spp., thus providing its biogeographic and evolutionary information. Our aim was to investigate the presence of *Anaplasma* spp. in pupal and adult *M. ovinus*.

## Methods

### Study areas and *M. ovinus* collection

In 2013, five *M. ovinus* specimens were collected during occasional tick sampling in South Xinjiang and were preserved in 70% ethanol. The sampling locations and time points were not recorded in detail.

In July 2016, 30 experimental specimens preserved in 70% ethanol were randomly selected from ~300 *M. ovinus* specimens collected from multiple sheep in Yahazhen of Kuqa in Aksu, Xinjiang (1029 m above sea level; 41°44'N, 83°14'E).

In June 2017, over 200 *M. ovinus* specimens were collected from each of the five sheep in Yahazhen of Kuqa in Aksu, Xinjiang. These *M. ovinus* specimens were placed in sampling vials with sufficient air and transported immediately to the laboratory for cryopreservation. Ten randomly selected *M. ovinus* specimens from each sheep and eight simultaneously collected pupal *M. ovinus* specimens from three sheep were regarded as experimental specimens.

In this study, 93 (5 + 30 + 50 + 8) samples were processed individually.

### DNA extraction, PCR of the *msp4* gene, and sequence analysis

The 70% ethanol-preserved *M. ovinus* specimens were washed twice with distilled water after being washed in a graded series of ethanol solutions with concentrations of 50%, 30% and 10%. The cryopreserved adult and pupal *M. ovinus* specimens were washed twice with distilled water for 1 h each.

Next, the genomic DNA of *M. ovinus* was extracted using the TaKaRa MiniBEST Universal Genomic DNA Extraction Kit Ver. 5.0 (Takara, Dalian, China, catalogue No. 9765). At the last step, the DNA sample was eluted twice with 50 μl of elution buffer, and the resultant 50 μl of genomic DNA was stored at -20 °C until use.

After that, the *msp4* gene of *Anaplasma* spp., which was PCR-amplified with the KOD-Plus amplification enzyme (Toyobo Co., Ltd., Osaka, Japan) and the Premix Taq^TM^ kit (TakaRa Taq^TM^ Version 2.0; Takara, catalogue No. R004A), was approximately 867 bp.

Each 50 μl PCR reaction mixture contained 25 μl of the 2× PCR solution for Premix Taq^TM^, 1 μl each of the forward and reverse primers (MSP4-F: 5'-GGG AGC TCC TAT GAA TTA CAG AGA ATT GTT TAC-3'; MSP4-R: 5'-CCG GAT CCT TAG CTG AAC AGG AAT CTT GC-3' [20, 22]), 1 μl of the DNA template, and distilled water.

The cycling conditions for the *msp4* gene amplification with primers MSP4-F and MSP4-R were as follows: initial denaturation at 94 °C for 5 min; 40 cycles at 94 °C for 30 s, 62 °C for 50 s, and 72 °C for 1 min; followed by final extension at 72 °C for 10 min.

All the amplicons were bidirectionally sequenced on an ABI PRISM^TM^ 3730XL DNA Analyzer (ABI, CarIsbad, America). The sequences were aligned with reference sequences downloaded from GenBank by means of MEGA 7.0 software. The sequences obtained in this study were deposited in the GenBank database under the accession numbers MG283274 and MG564176.

## Results

The *msp4* gene of *Anaplasma* spp. was PCR-amplified and sequenced in samples of the genomic DNA of adult and pupal *M. ovinus*. All five *M. ovinus* specimens collected in 2013 tested negative for *Anaplasma* spp., three out of the 30 *M. ovinus* specimens collected in 2016 tested positive for *Anaplasma* spp. with an identical *msp4* gene sequence, 20 (collected from two sheep) out of the 50 adult *M. ovinus* specimens collected in 2017 tested positive for *Anaplasma* spp., and five out of the eight non-blood-feeding pupal *M. ovinus* specimens collected in 2017 tested positive for *Anaplasma* spp. *Anaplasma* spp.-positive pupae were produced by adult *M. ovinus* from the two *Anaplasma* spp.-positive sheep, whereas three pupae from the other sheep tested negative for *Anaplasma* spp. There were no differences among the 21 *msp4* gene sequences (17 sequences from adult *M. ovinus* and four sequences from pupal *M. ovinus*) analyzed in 25 PCR amplicons.

**Fig. 2:**
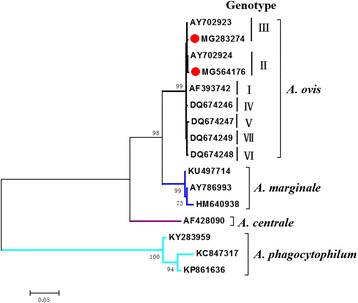
Molecular phylogenetic analysis of *A. ovis* strains by application of the ML method to the *msp4* gene sequence data. Evolutionary analyses were conducted in MEGA 7. Nucleotide sequence differences among the *msp4* gene sequences from different isolates of *A. ovis* confirmed seven genotypes [20]. Sequences of our specimens are marked with red circles

**Fig. 3:**
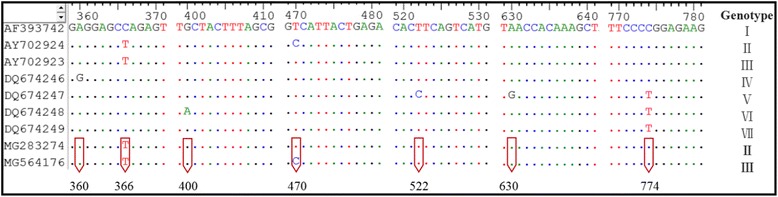
Nucleotide sequence differences among the *msp4* gene sequences from different isolates of *A. ovis*. The numbers represent the nucleotide positions starting at the translation initiation codon, adenine

Sequences of the two taxa obtained in this study having the highest similarity with the *msp4* gene sequence of *Anaplasma* spp. in the GenBank database are listed in Table 1, both of which are *A. ovis* isolates. The phylogenetic analysis of *msp4* confirmed that the obtained *Anaplasma* sp. was *A. ovis* (Fig. 2). Additionally, *A. ovis* isolates XJNJ (MG283274) and XJNJ2 (MG564176) were classified as *A. ovis msp4* Genotypes II and III based on A^360^T^366^G^400^C^470^T^522^A^630^C^774^ and A^360^T^366^G^400^T^470^T^522^A^630^C^774^, respectively (Fig. 3).

**Table 1:** Sequences relatively closest to the complete *msp4* gene sequence of *A. ovis* detected in the *M. ovinus* samples from South Xinjiang, China

Gene	GenBank ID	% sequence similarity (bp)	Remark
MG283274 (*A. ovis* isolate XJNJ)	*A. ovis* isolate KS9-b (KJ782401)	100 (852/852)	China: Xinjiang; 2012; sheep blood
	*A. ovis* isolate YC26 (KJ782404)	99 (851/852)	China: Xinjiang; 2012; sheep blood
	*A. ovis* isolate ATS20 (KJ782397)	99 (851/852)	China: Xinjiang; 2012; sheep blood
	*A. ovis* isolate Yongjing (HQ456347)	99 (851/852)	China: Yongjing County; 2010; sheep blood
	*A. ovis* isolate Italy 20 (KJ782401)	99 (851/852)	Italy: Sicily; 2004; ovine blood
MG564176 (*A. ovis* isolate XJNJ2)	*A. ovis* isolate MM9 (KY283958)	99 (851/852)	Turkey: Menemen, Izmir; 2011–2013; sheep blood
	*A. ovis* isolate Yuzhong (HQ456348)	99 (851/852)	China: Yuzhong County; 2010; sheep blood
	*A. ovis* isolate Italy 147 (AY702924)	99 (851/852)	Italy: Sicily; 2004; ovine blood
	A.ovis isolate 395 (KU497698)	99 (851/852)	Sudan; 2016; sheep
	*A. ovis* isolate Yuzhong (LC141088)	99 (850/852)	Mongolia; 2014; cattle blood

## Discussion

The presence of *A. ovis* DNA in adult and pupal *M. ovinus* collected in South Xinjiang, China, was confirmed by conventional PCR and sequencing. The sequence variation in the *msp4* gene among different *A. ovis* strains [20] confirmed that two genotypes of *A. ovis* were detected in this study.

The detection of *A. ovis* in *M. ovinus* has been reported previously [15]. *Anaplasma ovis* has also been discovered in adults of hippoboscid species (*Lipoptena cervi*), but not in the larvae and pupae [17]. *Bartonella* [4, 7], *Arsenophonus* and *Wolbachia* [7] can be transmitted vertically in *M. ovinus*. Both *M. ovinus* [4, 7, 17] and *L. cervi* mediate vertical transmission of *Bartonella* [29]. Nevertheless, the vertical transmission of *A. ovis via* parasites belonging to the family Hippoboscidae (Diptera: Hippoboscoidea) has not been reported. To the best of our knowledge, this study provides the first molecular evidence for the presence of *A. ovis* DNA in pupal *M. ovinus*. Additionally, the detection of *A. ovis* DNA in *M. ovinus* has not been reported in China. Our study suggests that *A. ovis* may be transmitted vertically *via M. ovinus*, and that *M. ovinus* may serve as a potential vector for *A. ovis*.

The diseases caused by *Anaplasma* spp. are a global issue, among which *A. ovis* causes ovine anaplasmosis. First discovered in sheep in 1912, *A. ovis* is currently widely distributed in Africa, Europe, Asia, and the USA [24, 25]. In China, *A. ovis* was first found in 1982 in the Xinjiang Uygur Autonomous Region, followed by Liaoning Province. Subsequent studies revealed that *A. ovis* is widely distributed in China and is particularly prevalent in the northwest region [22, 25]. *Anaplasma ovis* mainly parasitizes sheep, goats, wild ruminants [30, 31], cattle [28] and dogs [32]. Recently, an *A. ovis* variant was detected in a patient, indicating the zoonotic potential of this agent [33]. In addition, some sequences having the highest similarity with the *msp4* gene sequence of the two *M. ovinus*-derived *A. ovis* isolates in this study were detected in the blood of sheep sampled in Xinjiang in 2012. Taken together, Xinjiang has been seriously infested with *A. ovis*. It has been confirmed that various ticks, belonging to the genus *Ixodes*, serve as biological vectors for the transmission of *A. ovis* in China [22]. Our study confirmed the transmission of *A. ovis via M. ovinus* in China. Furthermore, currently there are seven *Anaplasma* spp. in China, including the recently discovered *A. capra* [26, 34, 35]. Additionally, vertical transmission of *A. ovis* was confirmed in the present study. Thus, *Anaplasma* spp. require close attention because the above-mentioned situations and phenomena lead to anaplasmosis in humans or animals and cause unpredictably huge economic losses.

## Conclusions

To our knowledge, this is the first report worldwide on the detection of *A. ovis* DNA in pupal *M. ovinus*, confirming the vertical transmission of *A. ovis* in *M. ovinus* and the potential of *M. ovinus* as the vector for *A. ovis*.

## Abbreviations

ML: Maximum-likelihood
PCR: Polymerase chain reaction
